# Inverted reamer technique for bone grafting of the acetabulum: technical note

**DOI:** 10.1186/s13018-021-02810-x

**Published:** 2021-10-30

**Authors:** Yuki Okutani, Hiroshi Fujita, Hideto Harada, Masanao Kataoka, Yu Shimizu, Yoshiki Murotani

**Affiliations:** 1 Department of Orthopaedic Surgery, Kyoto Katsura Hospital, Kyoto, Japan; 2 Center for Hip and Knee Arthroplasty, Department of Orthopaedic Surgery, Kyoto Rakuyo Hospital, Iwakura-Agura-cho 143, Sakyo-ku, Kyoto, 606-0017 Japan; 3 Department of Orthopaedic Surgery, Kyoto City Hospital, Kyoto, Japan; 4 Department of Orthopaedic Surgery, Graduate School of Medicine, Kyoto University, Kyoto, Japan

**Keywords:** Total hip arthroplasty, Cement, Bone graft, Inverted reamer, Dysplasia

## Abstract

**Background:**

Socket fixation with bone grafting for dysplastic hips is technically demanding, and inadequate coverage of the socket may cause poor results in patients with severely dysplastic hips. An accurate technique to form a bone graft to fit into the defect is necessary. We aim to introduce the simple method of bone grafting, “inverted reamer technique” in cemented total hip arthroplasty (cTHA).

**Methods:**

After acetabular preparation with a normal acetabular reamer, the bone graft was prepared from the resected femoral head with the inverted reamer. The graft can be press-fit into the defect of the acetabulum with good compatibility through this method. Then, the bone graft was fixed with 1–3 screws and the socket was implanted with bone cement.

**Results:**

The “inverted reamer technique” can easily and automatically create a well-fit graft. This method is simple and technically less demanding; it can be performed by every surgeon, including trainee and inexperienced surgeons.

**Conclusion:**

This method can improve the outcome of cTHA for dysplastic hips by preserving bone stock and increasing bone coverage of the socket implanted in the anatomic position.

## Introduction

Total hip arthroplasty (THA) is one of the most successful treatments for hip dysfunction, and many excellent outcomes have been previously reported with this technique [1–5]. THA is divided into three types according to the fixation method: cemented, hybrid, or cementless. For cemented THA (cTHA), some studies have shown in comparison with problems of the femoral stem, socket problems more frequently require revision procedures [5–8]. This outcome might be improved by the development of highly crosslinked polyethylene (HXLPE), which reduces polyethylene wear [9, 10]. Moreover, robust socket fixation is sometimes difficult, especially in dysplastic hips, because of the massive bony defect of the acetabulum. Thus, successful cTHA requires an improved socket-fixation technique. Socket fixation with bone grafting for dysplastic hips is technically demanding, and inadequate coverage of the socket may cause poor results in patients with severely dysplastic hips [11]. Bone grafting can increase bone stock, improving the bone coverage of the socket and facilitating cement pressurization. However, a previous report showed that the 11.8-year incidence of loosening of the acetabular socket with bone grafting was high for dysplastic hips, and bone grafting for dysplastic hips is only recommended in cases with a massive bone defect [12]. In contrast, some other reports have shown good outcomes with bone grafting [13–15]. To ensure successful bone grafting, an accurate technique to form a bone graft to fit into the defect is necessary: however, this step is extremely technically demanding. Thus, we developed a new simpler method, in which the curvature of the bone graft is matched to that of the bone defect on the roof of the acetabulum using inverted reamers, which were introduced for cartilage removal of a femoral head [16, 17], such that the bone graft is well-fitted to the roof of the acetabulum. As a result, bone grafting is achieved mechanically, easily, and quickly. In this note, we will introduce this simple method of bone grafting.

## Method

**Fig. 1:**
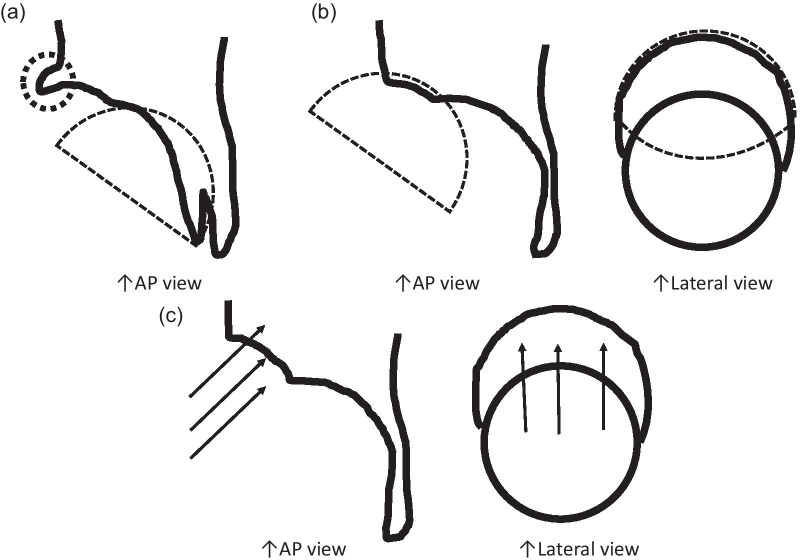
Schema for preparation of the acetabulum for bone grafting and socket fixation. **a** Preparation of the acetabulum; reaming of the acetabulum and removal of the osteophytes. **b** Preparation of the roof of the acetabulum; reaming of the acetabulum with a normal reamer. **c** Preparation of the roof of the acetabulum; creation of some drill holes on the roof. *AP* anteroposterior

Bone defects are usually recognized on the roof of the acetabulum. These defects necessitate the creation of a bone graft to fit into the defect by trimming with instruments, including a bar or bone luer. However, a tight, stable fit of the graft with may be difficult to achieve with this conventional method. As an alternative, our method allows easy creation of a bone graft that is a good fit for the defect. The “inverted reamer technique” is described in more detail below:

1. Indications for bone grafting for the acetabulum
  The need for bone grafting is determined during the operation. At our institute, bone grafting of the acetabulum is performed when the height between the lateral-cranial edge of the acetabulum and the lateral edge of the socket is more than 10 mm after reaming the acetabulum until the socket with the preoperatively planned size is set just on the transverse ligament (this procedure is called “anatomical placement”).
2. Surgical approach to the acetabulum
  At our institute, cTHA is performed with a modified Dall’s approach in the lateral decubitus position [18, 19]. The cTHA procedure has been described briefly in a previous report [20]. The entire acetabulum is visualized with a pin-retractor placed in the ilium and a horizontal retractor.
3. Acetabulum preparation
  The acetabulum is reamed until the sclerotic bone plate is removed and trabecular bone with fresh bleeding is recognized (Fig. 1a, 2a). Osteophytes and capsules around the acetabulum are removed completely in order to observe the true acetabulum and determine the graft size. The roof of the acetabulum, where the bone graft is eventually transplanted, is reamed by a normal acetabular reamer. Consequently, the sclerotic bone is removed, and the cancellous bone, which is active and vascular, is exposed. At this point, the reamer should be stabilized to obtain a complete spherical shape on the roof considering the curvature (Fig. 1b, 2b). After reaming, drill holes with diameters of 2.5 and 6 mm are created to vascularize the bony bed for transplantation. (Fig. 1c, 2c). The approximate size of the bone graft is checked with the trial socket.
4. Bone graft preparation
  The inverted reamers (Femoral Head Reamer Set; Spierings Orthopaedics BVS, Nijmegen, Netherlands) are prepared (Fig. 3a). The inverted reamer is hemispherical, and unlike a normal reamer with blades located on the outer surface, the blades of the inverted reamer are located on the internal side. After fixing the resected femoral head on a bone fixator, the head is cut into one-third or half, depending on the size of the bony defect, to create a piece of bone. A weight-loaded (proximo-medial) part of the head is cut and trimmed for the graft. Then, the piece is reamed by the inverted reamer. The inverted reamer is 2 mm larger than the normal reamer that had been used for the roof of the acetabulum (this is described later in more detail). The surface of the trabecular bone is reamed by the inverted reamer to prepare a convex surface. The sclerotic cortical bone on the articular surface is preserved (Fig. 3b, 4). At grafting, the convex surface of the trabecular bone is placed on the medial side for attachment to the acetabulum, and that of the sclerotic bone is placed on the lateral side.
5. Bone transplantation and socket implantation
  After preparation, the piece can be press-fit into the defect of the acetabulum with good compatibility. Then, minced bone obtained during reaming of the acetabulum is applied on the surface of the trabecular bone of the piece (The side with minced bone will be medial and attached to the acetabulum). The piece with the minced bone is placed on the defect and fixed by two or three 2.0-mm Kirschner wires (K-wires) (Fig. 5a, 6a). Next, 1–3, 4.5-mm bioabsorbable screws made of l-polylactic acid (Superfixorb; TEIJIN MEDICAL TECHNOLOGY CO., Osaka, Japan) are inserted with a washer instead of the K-wire one after another. When inserting the screws, the K-wires should be carefully bent to the lateral sides because straight K-wires will obstruct screw insertion (Fig. 5b, 6b). Because an inferior part of the bone graft can be trimmed for socket fixation, these screws should be placed in the superior part of the graft. After inserting the screws, the acetabulum is reamed and trimmed for socket implantation again while taking care of the fixed bone graft (Fig. 6c). Then, the HXLPE non-flanged socket (STD socket; Kyocera Medical, Osaka, Japan) is implanted with polymethylmethacrylate (PMMA) bone cement (Fig. 5c, 6d). After the cement has hardened, all K-wires are carefully removed. Finally, the residual osteophytes around the socket, which may cause impingement and dislocation, are resected.
6. Postoperative rehabilitation
  On the first postoperative day, the patients are allowed full weight-bearing dependent on their pain. Restrictions in weight-bearing are not required with this technique.

**Fig. 2:**
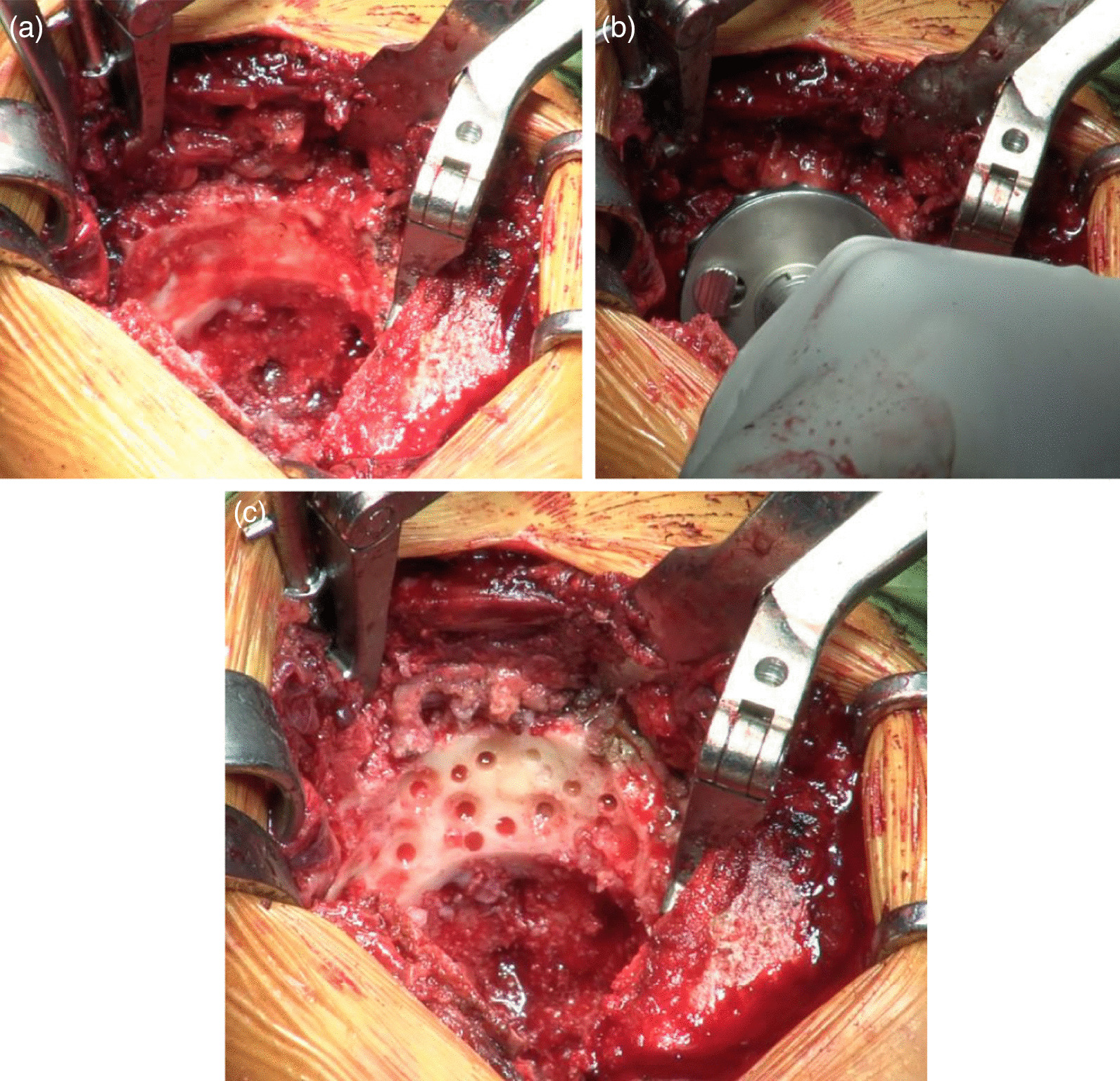
Photographs showing preparation of the acetabulum for bone grafting and socket fixation. **a** The original acetabulum was reamed, and some anchoring holes were created. **b** The roof was reamed with a normal reamer. **c** Some drill holes were created on the roof

**Fig. 3:**
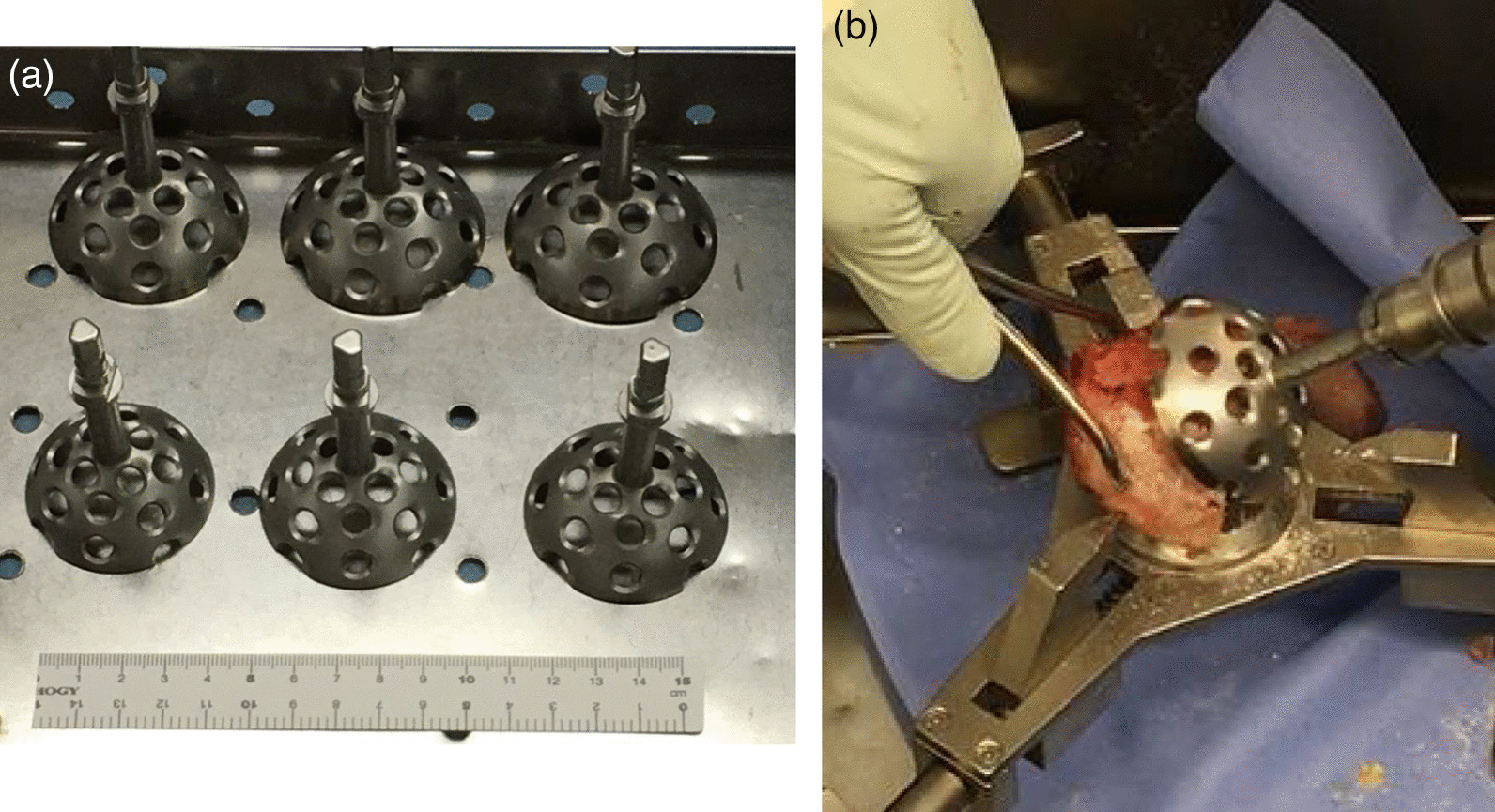
Photographs showing the inverted reamer and shaving of the bone graft from the resected femoral head. **a** A photograph of the inverted reamer. **b** A piece of the femoral head was fixed on a bone fixator and reamed with the inverted reamer. The graft was shaved from the piece

**Fig. 4:**
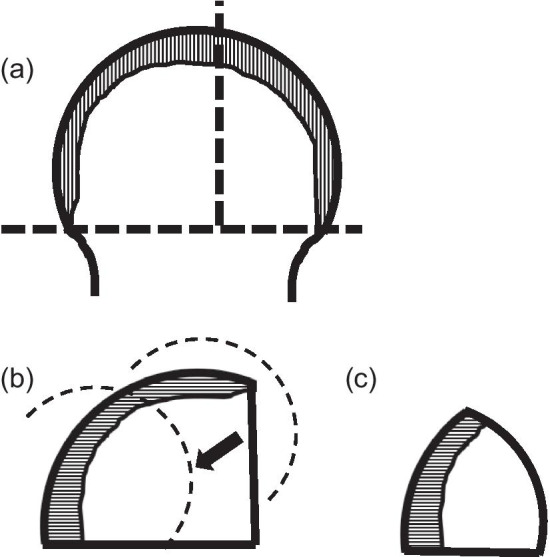
Shaving of the bone graft from the resected femoral head. **a** The resected femoral head was cut into an adequate size. **b** The side of the trabecular bone in the piece of the femoral head was reamed with the inverted reamer, and extra bone was removed with a leur. The black arrow indicates the movement of the inverted reamer. **c** A piece with a convex surface was created. This piece will be transplanted onto the roof of the acetabulum. Sclerotic bone is shown as a stripe pattern. The dotted line is the cut line

**Fig. 5:**
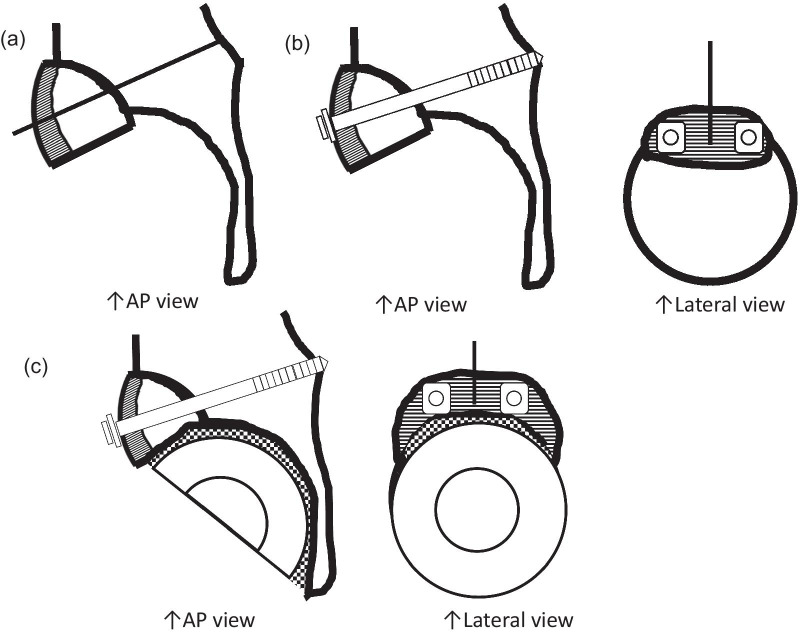
The schema of transplantation of the bone graft and the socket. **a** The piece created with the inverted reamer was temporarily fixed with K-wires. **b** The piece was fixed by insertion of the bioabsorbable screws (Superfixorb®). **c** The socket was fixed by cement. The K-wire was removed after the cement hardened. Sclerotic bone is shown as a stripe pattern. Cement is shown as the check pattern

**Fig. 6:**
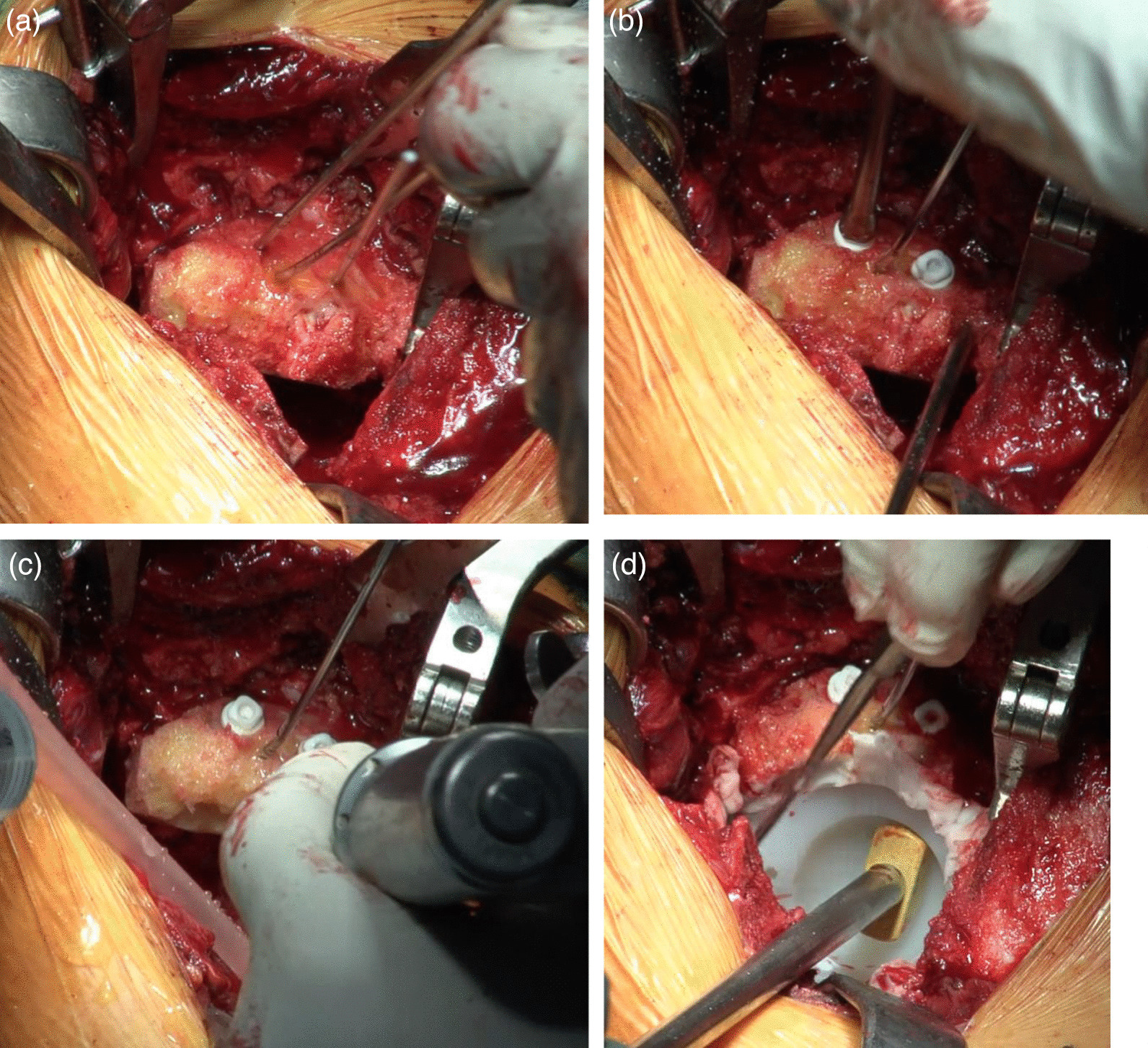
Photographs showing transplantation of the bone graft and socket. **a** The piece created with the inverted reamer was temporarily fixed with K-wires. **b** The piece was fixed by insertion of bioabsorbable screws (Superfixorb®). **c** The acetabulum was reamed again for socket fixation. **d** The socket was fixed by cement. The K-wire was removed after the cement hardened

## Case presentation

**Fig. 7:**
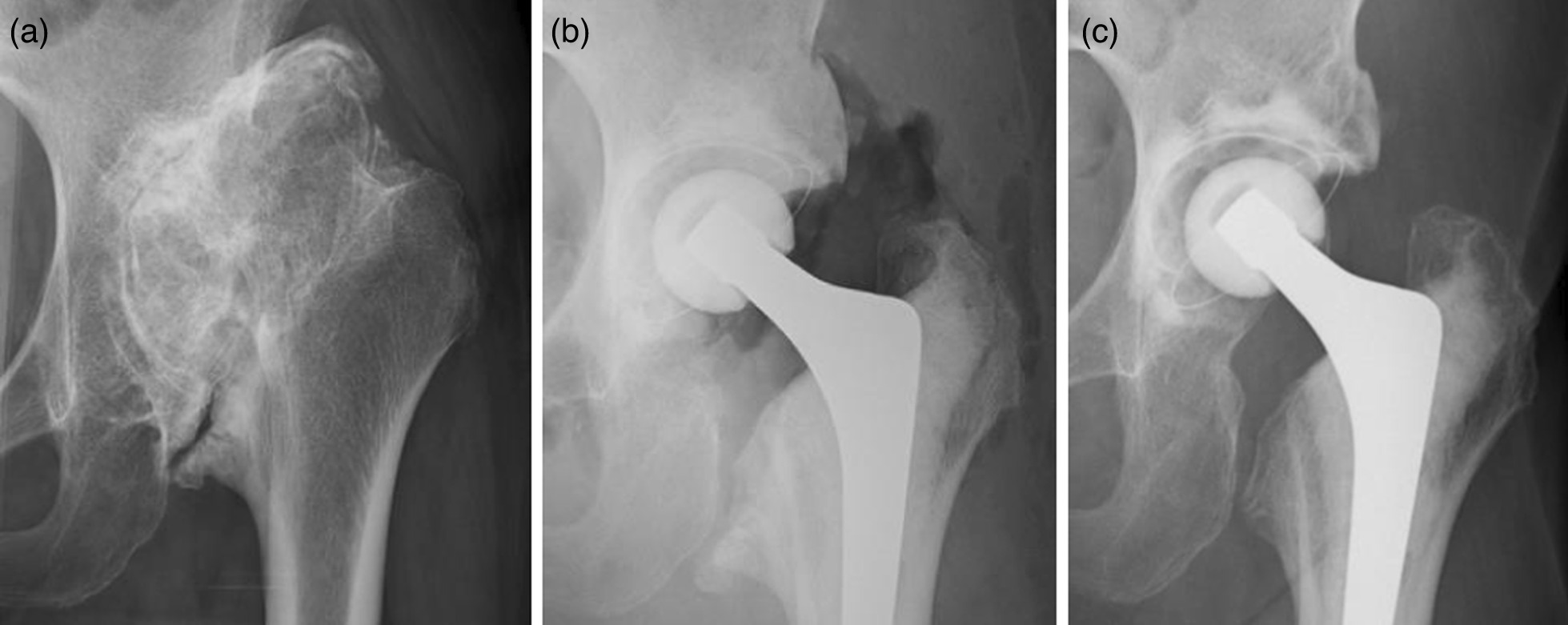
AP radiographs of the patient who underwent cTHA via this method. **a** Preoperative AP radiograph. **b** Postoperative AP radiograph. Two Superfixorb screws, 35 mm and 45 mm, were used. **c** AP radiograph at 2 years of follow-up

A 71-year-old woman underwent cTHA for secondary osteoarthritis (OA) with a dysplastic hip. The preoperative anteroposterior (AP) radiograph is shown in Fig. 7a. Her dysplastic hip was classified as Group III according to Crowe’s classification [21]. The acetabulum was reamed with a 46-mm normal reamer, and the roof of the acetabulum was reamed with a 48-mm normal reamer. The bone graft was created using a 50-mm inverted reamer. The graft was fixed with two Superfixorb screws. The head was 28 mm and the socket was 44 mm in diameter. The postoperative AP radiograph is shown in Fig. 7b. The AP radiograph at 2 years of follow up is shown in Fig. 7c. The bone coverage of the socket increased from 151° to 180° using this technique. The patient was allowed to walk with full weight-bearing on the first postoperative day.

## Discussion

The present study has introduced a method to perform bone grafting using an inverted reamer. This method is useful because it allows surgeons to create the graft mechanically, which fits well into the defect. Moreover, bone graft creation with this method is faster than that with the conventional method, in which surgeons create bone grafts manually using a leur or a rotational burr. A previous report showed that the extent of socket coverage prevented late failure of the socket [22]. Thus, this method may improve the outcome of THA.

A report published in a German journal described a similar method along with the outcomes obtained in 55 hips [23]. However, only the abstract of that report is available in English. There are some differences between our proposed method and the method described in that report. First, the previous report does not clearly describe the relationship between the size of a normal reamer and that of an inverted reamer, while the inverted reamer is 2 mm larger than the normal reamer in our method. Second, the previous method uses no augmentation, including screws, routinely, while our method uses a couple of screws. Third, in the previously described method, postoperative non-weight bearing is recommended when the graft is larger than 10 mm, while full-weight bearing is allowed in our method. Fourth, in the previous method, the articular cartilage was removed by an inverted reamer and the reamed surface was attached to the acetabulum to match the bony trabecular structure in the direction of weight-loading. In contrast, in our method, the unreamed surface (sclerotic bone) is placed on the lateral side, not attached to the acetabulum. To our knowledge, no other reports in the English literature have described a similar method.

A bioabsorbable screw with a washer was employed for graft fixation at our institute. The washer dissipates the pressure from the screw head and prevents the bone graft from being crushed. As an additional benefit, since the screw is bioabsorbable, it does not need to be removed during revision surgery. A previous report using a screw for a bone graft showed a good outcome [24]. Thus, these screws might show as much strength as a metal screw for fixation. Metal cancellous screws, including a cannulated screw, can also be used instead of the bioabsorbable screw in this method.

Five methods have been reported for dysplastic hips: proximal positioning of the socket, customized acetabular augments, combination with trabecular metal, bulk bone grafting on the roof of the acetabulum, and impaction bone grafting [25, 26]. We advocated that bulk bone grafting is the best in terms of the bone stock and cup position. Kobayashi et al. presented a technique to enable the original anatomic placement of the cemented socket with a bulk bone autograft [27]. Some reports have shown excellent results of cTHA with acetabular bulk bone grafting [13–15]. In terms of the strength of the abductor muscle and long-term durability, anatomic placement of the socket is better, although there are objections to this approach [28, 29]. Therefore, the achievement of anatomic placement of the socket with adequate bone grafting via our method may improve the outcome of cTHA for dysplastic hips.

This method involves three ingenuities. First, we applied minced bone on the graft. The minced bone can fill the gap to improve the stability of the graft and the osteosynthesis between the acetabulum and the graft, and also prevent cement intrusion into the gap between the graft and the host bone. Second, we reamed the graft using a 2-mm-larger inverted reamer. This allowed us to create a bone graft with a slightly larger diameter than the diameter of the defect and stabilized the graft in the defect by a press-fit mechanism. Third, the surface of the resected femoral head was utilized. The trabecular bone of the head was directed to the roof of the acetabulum because the trabecular bone is more bioactive for bone healing with the acetabulum. In contrast, the side of the sclerotic bone of the head was directed to the lateral side, since sclerotic bone may have more strength to endure loading by screw fixation. This technique was useful because it could yield good and robust stability immediately after fitting the graft on the roof of the acetabulum by matching the curvature.

However, this method has two drawbacks. First, we fixed the graft using a couple of screws. This procedure is time-consuming and might cause massive bleeding by damaging the intrapelvic vessels. Second, a larger exposure of the roof of the acetabulum is necessary for grafting. This may be difficult in patients with a small build and might damage a branch of the superior gluteal artery.

## Conclusions

The “inverted reamer technique” introduced in this report is useful because it can easily and automatically create a well-fit graft. This method is simple and technically less demanding; it can be performed by every surgeon, including trainee and inexperienced surgeons. This method can improve the outcome of cTHA for dysplastic hips by preserving bone stock and increasing bone coverage of the socket implanted in the anatomic position.

## Data Availability

These are available according to reader’s request.

## Abbreviations

THA: Total hip arthroplasty
cTHA: Cemented total hip arthroplasty
HXLPE: Highly cross-linked polyethylene
K-wire: Kirschner wire
PMMA: Polymethylmethacrylate
AP: Anteroposterior
